# Medicines

**Published:** 1876-03

**Authors:** E. H. Gibbs


### *MEDICINES.*

BY E. H. GIBBS, A.M., A.D.

We have always been opposed to giving medicines, unless imperatively demanded by the condition of the patient. All cathartics debilitate during their operation. The use of stimulating drugs is usually followed by debility and distress for days afterward; and the wise physician will always carefully consider every case, weighing the evil certain to result against the benefits to be reasonably hoped for from medication. In a great majority of cases to be met with in private practice, no medicine should be given. The system often becomes clogged by excesses in eating or drinking, or its action is impaired by some accidental cause, such as over-fatigue or taking cold. Rest and careful nursing are the best remedies in such cases. But there are cases of a more aggravated character, that require the prompt and skillful attention of the educated physician. Such are cases that have gone beyond the recuperative power of nature, and demand the most decided treatment. Inflammatory cases are of this character. In those that attack the vital organs the life of the patient depends on the skill of the physician. The most fatal of all diseases are those known as bowel complaints of children, which carry off thousands of young children every summer.

It is a great mistake to suppose that these diseases are almost exclusively confined to the large cities. They are just as prevalent and just as fatal in country villages and in the rural districts, and as often assume the severe type of "cholera infantum." There is, unfortunately, a diversity of opinion among physicians as to the nature of this disease and its proper treatment. The most popular medical authorities describe it as a disease of the bowels, and their principal remedies always consist of some preparation of opium. We will relate, briefly, how we came to renounce forever this view of the pathology of cholera infantum, or summer complaint of children, and its treatment by means of "soothing syrups" and other poisonous preparations of opium. Twelve years ago last summer, while making a voyage at sea, we were called upon to treat a child eighteen months old who had been suffering with diarrhœa for several days, and seemed to be getting worse. We employed the *usual* remedies, which promised the *usual* results. The diarrhœa was checked, and then came, *as a consequence*, threatenings of congestion of the brain and lungs from an accumulation of fluids in the bowels, for which there was no other avenue of escape. The parents had lost three children of the same disease, and they became almost frantic with grief at what to all of us seemed the almost certain prospect of losing their only surviving child. Nothing appeals so forcibly to the sympathies of the physician as the sufferings of a helpless little child whose life is in his hands.

We stood gazing at the little patient and his suffering parents, wondering if we had done all that could be done to save their darling, when a gentleman with whom we had become acquainted on the voyage, called us aside. With many apologies for his interference, as he was not a physician, he informed us that he always carried with him during the summer months a remedy which he had found infallible in such cases, and asked if we would give it a trial. The offer was gladly accepted, although we had very little faith in its power at that stage of the disease. We, however, gave it as directed and awaited the result. To our surprise and very great joy there was a marked improvement in the symptoms within three hours, and the child was out of danger within twenty-four hours.

From that day to this we have always kept on hand an ample supply of this medicine, and have *never* since had a fatal case of cholera infantum or other bowel complaint of children. Last summer, when we volunteered our services to aid in arresting the fearful mortality among children from this terrible disease, of the 230 cases treated we are not aware that there was a single fatal case. And still, of the fifty physicians who presented themselves at our office in response to an appeal through the New York *Herald* for aid, *not one* approved our plan of treatment, or would join us in the good work. Such is the result of that blind adherence to theory which no evidence can shake.

The fatal error that nine physicians in ten make is in supposing that what are called "bowel complaints" of children are diseases of the bowels, when, in fact, the seat of the disease is in the liver, and it *always*

yields readily to the appropriate treatment. Some time or other—it may be in ten years, or in one hundred years—*all educated* physicians will recognize this fact, and then fatal "bowel diseases of children" will be comparatively unknown.

